# Current use of vasopressors in septic shock

**DOI:** 10.1186/s13613-019-0498-7

**Published:** 2019-01-30

**Authors:** Thomas W. L. Scheeren, Jan Bakker, Daniel De Backer, Djillali Annane, Pierre Asfar, E. Christiaan Boerma, Maurizio Cecconi, Arnaldo Dubin, Martin W. Dünser, Jacques Duranteau, Anthony C. Gordon, Olfa Hamzaoui, Glenn Hernández, Marc Leone, Bruno Levy, Claude Martin, Alexandre Mebazaa, Xavier Monnet, Andrea Morelli, Didier Payen, Rupert Pearse, Michael R. Pinsky, Peter Radermacher, Daniel Reuter, Bernd Saugel, Yasser Sakr, Mervyn Singer, Pierre Squara, Antoine Vieillard-Baron, Philippe Vignon, Simon T. Vistisen, Iwan C. C. van der Horst, Jean-Louis Vincent, Jean-Louis Teboul

**Affiliations:** 1 Department of Anesthesiology, University Medical Center Groningen, University of Groningen, Hanzeplein 1, P.O. Box 30.001, 9700RB Groningen, The Netherlands; 2 New York University Medical Center, New York, USA; 3 Columbia University Medical Center, New York, USA; 4 Erasmus MC University Medical Center, Rotterdam, Netherlands; 5 Pontificia Universidad Católica de Chile, Santiago, Chile; 6 Department of Intensive Care, CHIREC Hospitals, Université Libre de Bruxelles, Brussels, Belgium; 7 Department of Intensive Care Medicine, School of Medicine Simone Veil, Raymond Poincaré Hospital (APHP), University of Versailles-University Paris Saclay, 104 boulevard Raymond Poincaré, 92380 Garches, France; 8 Département de Médecine Intensive-Réanimation et de Médecine Hyperbare, Centre Hospitalier Universitaire Angers, Institut MITOVASC, CNRS, UMR 6214, INSERM U1083, Angers University, Angers, France; 9 Department of Intensive Care, Medical Centre Leeuwarden, Leeuwarden, The Netherlands; 10 Department of Anaesthesia and Intensive Care Units, Humanitas Research Hospital and Humanitas University, Milan, Italy; 11 Cátedra de Farmacología Aplicada, Facultad de Ciencias Médicas, Universidad Nacional de La Plata y Servicio de Terapia Intensiva, Sanatorio Otamendi, Buenos Aires, Argentina; 12 Department of Anesthesiology and Intensive Care Medicine, Kepler University Hospital and Johannes Kepler University Linz, Linz, Austria; 13 Assistance Publique des Hopitaux de Paris, Department of Anaesthesia and Intensive Care, Hôpitaux Universitaires Paris-Sud, Université Paris-Sud, Hôpital de Bicêtre, Le Kremlin-Bicêtre, France; 14 Section of Anaesthetics, Pain Medicine and Intensive Care, Imperial College London, London, UK; 15 Assistance Publique-Hôpitaux de Paris Paris-Sud University Hospitals, Intensive Care Unit, Antoine Béclère Hospital, Clamart, France; 16 Departamento de Medicina Intensiva, Facultad de Medicina, Pontificia Universidad Católica de Chile, Santiago, Chile; 17 Assistance Publique Hôpitaux de Marseille, Service d’Anesthésie et de Réanimation CHU Nord, Aix Marseille Université, Marseille, France; 18 Service de Réanimation Médicale Brabois et pôle cardio-médico-chirurgical, CHRU, INSERM U1116, Université de Lorraine, Brabois, 54500 Vandoeuvre les Nancy, France; 19 Department of Anesthesia, Burn and Critical Care, APHP Hôpitaux Universitaires Saint Louis Lariboisière, U942 Inserm, Université Paris Diderot, Paris, France; 20 Assistance Publique-Hôpitaux de Paris, Paris-Sud University Hospitals, Medical Intensive Care Unit, Bicêtre Hospital, Le Kremlin-Bicêtre, France; 21 INSERM UMR_S 999, Paris-Saclay University, Le Plessis-Robinson, France; 22 Department of Cardiovascular, Respiratory, Nephrological, Anesthesiological and Geriatric Sciences, University of Rome “La Sapienza”, Rome, Italy; 23 INSERM 1160 and Hôpital Lariboisière, APHP, University Paris 7 Denis Diderot, Paris, France; 24 Queen Mary University of London, London, UK; 25 Department of Critical Care Medicine, University of Pittsburgh, Pittsburgh, USA; 26 Institut für Anästhesiologische Pathophysiologie und Verfahrensentwicklung, Universitätsklinikum, Ulm, Germany; 27 Department of Anesthesiology and Intensive Care Medicine, Rostock University Medical Centre, Rostock, Germany; 28 Department of Anesthesiology, Center of Anesthesiology and Intensive Care Medicine, University Medical Center Hamburg-Eppendorf, Hamburg, Germany; 29 Department of Anesthesiology and Intensive Care, Uniklinikum Jena, Jena, Germany; 30 Bloomsbury Institute of Intensive Care Medicine, Division of Medicine, University College London, London, UK; 31 ICU Department, Réanimation CERIC, Clinique Ambroise Paré, Neuilly, France; 32 Assistance Publique-Hôpitaux de Paris, Intensive Care Unit, University Hospital Ambroise Paré, Boulogne-Billancourt, France; 33 INSERM U-1018, CESP, Team 5, University of Versailles Saint-Quentin en Yvelines, Villejuif, France; 34 Medical-Surgical Intensive Care Unit, INSERM CIC-1435, Teaching Hospital of Limoges, University of Limoges, Limoges, France; 35 Institute of Clinical Medicine, Aarhus University, Palle Juul-Jensens Boulevard 99, 8200 Aarhus N, Denmark; 36 Department of Critical Care, University Medical Center Groningen, University of Groningen, Hanzeplein 1, P.O. Box 30.001, 9700 RB Groningen, The Netherlands; 37 Department of Intensive Care, Erasme University Hospital, Université Libre de Bruxelles, Brussels, Belgium; 38 Service de Réanimation Médicale, Hôpital de Bicêtre, Hôpitaux Universitaires Paris-Sud, Le Kremlin-Bicêtre, France

**Keywords:** Shock, Sepsis, Septic shock, Resuscitation, Vasopressor, Vasoactive agonists, Norepinephrine, Arterial blood pressure

## Abstract

**Background:**

Vasopressors are commonly applied to restore and maintain blood pressure in patients with sepsis. We aimed to evaluate the current practice and therapeutic goals regarding vasopressor use in septic shock as a basis for future studies and to provide some recommendations on their use.

**Methods:**

From November 2016 to April 2017, an anonymous web-based survey on the use of vasoactive drugs was accessible to members of the European Society of Intensive Care Medicine (ESICM). A total of 17 questions focused on the profile of respondents, triggering factors, first choice agent, dosing, timing, targets, additional treatments, and effects of vasopressors. We investigated whether the answers complied with current guidelines. In addition, a group of 34 international ESICM experts was asked to formulate recommendations for the use of vasopressors based on 6 questions with sub-questions (total 14).

**Results:**

A total of 839 physicians from 82 countries (65% main specialty/activity intensive care) responded. The main trigger for vasopressor use was an insufficient mean arterial pressure (MAP) response to initial fluid resuscitation (83%). The first-line vasopressor was norepinephrine (97%), targeting predominantly a MAP > 60–65 mmHg (70%), with higher targets in patients with chronic arterial hypertension (79%). The experts agreed on 10 recommendations, 9 of which were based on unanimous or strong (≥ 80%) agreement. They recommended not to delay vasopressor treatment until fluid resuscitation is completed but rather to start with norepinephrine early to achieve a target MAP of ≥ 65 mmHg.

**Conclusion:**

Reported vasopressor use in septic shock is compliant with contemporary guidelines. Future studies should focus on individualized treatment targets including earlier use of vasopressors.

## Background

Circulatory shock affects about one-third of patients admitted to intensive care [1] and is associated with increased mortality rates [1–3]. Four pathophysiological mechanisms of shock (i.e., distributive, hypovolemic, cardiogenic, and obstructive) have been distinguished [3, 4], which can be present alone or in combination [5]. In patients requiring vasopressor therapy, the majority are diagnosed as having septic shock (62%), followed by cardiogenic and hypovolemic shock (both 16%), and other types of distributive shock (4%) and obstructive shock (2%) [6]. In this work, we focused on septic shock, as the most common form of distributive shock.

The essential step in the management of patients with septic shock is to increase systemic and regional/microcirculatory flow. Increasing arterial blood pressure (ABP) with vasopressors when patients are hypotensive is used to improve the input pressure driving organ perfusion. However, except for the choice of the first-line agent (norepinephrine), there is no clear consensus regarding the use of vasopressors in septic shock. For instance, for life-threatening sepsis-induced hypotension, the 2012 Surviving Sepsis Campaign (SSC) guidelines recommended early initiation of norepinephrine in patients with low diastolic blood pressure (as marker of low arterial tone) [7]. However, the most recent 2016 SSC guidelines are less precise about the appropriate time to initiate norepinephrine [8] so the question about optimal timing remains. The guidelines recommend a mean arterial pressure (MAP) of at least 65 mmHg should be used as an initial target value [8] and that vasopressors should be started immediately if patients remain hypotensive during or after fluid resuscitation (strong recommendation, moderate quality of evidence) [9]. Higher targets should be considered in patients with chronic arterial hypertension, although this remains controversial [2, 8, 10]. However, some data suggest that individualization of the MAP target alone may not improve outcome [11], so other measures should be considered to increase systemic blood flow. Furthermore, it is still a matter of debate whether vasopressin or other agents should be added to norepinephrine in cases of refractory hypotension [12]. Vasopressin use may be associated with a lower risk of atrial fibrillation and mortality [13]. Finally, information on vasopressor tolerance, side effects, and potential effects on cardiac function is scarce.

Therefore, hemodynamic management of early septic shock is a perpetual work in progress with unresolved questions and low quality of evidence [14], and further research on the optimal use of vasopressors is needed. Yet, to aid the design and interpretation of future studies, it is imperative to establish a knowledge base of what can be considered standard of care. We thus aimed to evaluate current practice, preferences, and therapeutic goals on the use of vasopressor drugs in the treatment of patients with septic shock. Furthermore, based on the answers, we identified areas of interest for which we approached international experts in the field for their opinions/recommendations.

## Methods

A survey was developed by the Cardiovascular Dynamics Section of the European Society of Intensive Care Medicine (ESICM). The survey consisted of 27 questions on the use of vasoactive drugs. This article focuses on 17 questions related to the use of vasopressors in septic shock, defined as persistent hypotension despite fluid resuscitation [15–17]. These were organized into two main sections: (1) the profile of respondents and their centers (Table 1) and (2) triggering factors, first-line drug choice, dosing, timing, targets, additional treatment strategies, and effects of vasopressors (Table 2).

**Table 1:** Baseline characteristics of survey respondents

	Response rate		
	Total	Europe	Outside Europe
Valid respondents	839 (100%)	546 (65%)	293 (35%)
Main specialty area			
Intensive care	545 (65%)	313 (57%)	232 (79%)
Anesthesiology	197 (23%)	164 (30%)	33 (11%)
Internal medicine	53 (6%)	44 (8%)	9 (3%)
Surgery	8 (1%)	3 (0.5%)	5 (2%)
Other	36 (4%)	22 (4%)	14 (5%)
Experience as intensivist			
Full time > 5 years	445 (53%)	282 (52%)	163 (56%)
Full time 2–5 years	98 (12%)	49 (9%)	49 (17%)
Full time < 2 years	46 (5%)	26 (5%)	20 (7%)
Part time intensivist	141 (17%)	116 (21%)	25 (9%)
Not specialized (yet)	108 (13%)	73 (13%)	35 (12%)
Type of institution			
University hospital	353 (42%)	262 (48%)	91 (31%)
Non-university public hospital	183 (22%)	149 (27%)	34 (12%)
University affiliated hospital	178 (21%)	100 (18%)	78 (27%)
Private hospital	113 (13%)	31 (6%)	82 (28%)
Other	12 (1%)	4 (1%)	8 (3%)
Type of ICU			
Mixed ICU	627 (75%)	408 (75%)	219 (75%)
Surgical ICU	88 (10%)	68 (12%)	20 (7%)
Medical ICU	83 (10%)	50 (9%)	33 (11%)
Other	41 (5%)	20 (4%)	21 (7%)
Number of ICU beds			
≤ 5	23 (3%)	16 (3%)	7 (2%)
6–10	221 (26%)	176 (32%)	45 (15%)
11–15	188 (22%)	135 (25%)	53 (18%)
16–20	150 (18%)	89 (16%)	61 (21%)
≥ 20	257 (31%)	130 (24%)	127 (43%)
Number of patients admitted per year			
< 500	188 (22%)	135 (25%)	53 (18%)
500–1000	291 (35%)	193 (35%)	98 (33%)
1001–1500	178 (21%)	115 (21%)	63 (22%)
1501–2000	92 (11%)	58 (11%)	34 (12%)
> 2000	90 (11%)	45 (8%)	45 (15%)

**Table 2:** Survey questions and answers on vasopressor use in septic shock

	Respondents
	No (%)
How do you measure arterial blood pressure in septic shock?	
Always invasively and continuously via an arterial line	707 (84%)
Invasively only in case of severe shock	97 (12%)
Mostly non-invasively and discontinuously (arm cuff)	32 (4%)
Mostly non-invasively but continuously using applanation tonometry	2 (0.3%)
Mostly non-invasively but continuously using finger cuff	1 (0.1%)
What is your main triggering factor(s) for initiating a vasopressor in septic shock?	
A low diastolic blood pressure whatever the correction of hypovolemia	29 (3%)
Insufficient cardiac output response to the initial fluid resuscitation	56 (7%)
Insufficient central venous oxygen saturation response to the initial fluid resuscitation	16 (2%)
Insufficient mean arterial pressure response to the initial fluid resuscitation	700 (83%)
Other	38 (5%)
What is your first line vasopressor in the treatment of hypotension?	
Adrenaline/epinephrine	4 (0.5%)
Dopamine	17 (2%)
Noradrenaline/norepinephrine	816 (97%)
Vasopressin/terlipressin	2 (0.3%)
Phenylephrine	0 (0%)
When do you use your vasopressor?	
I try to avoid any use of vasopressors and stick to volume therapy	15 (2%)
I use a vasopressor early, before complete volume resuscitation (despite preload dependency)	104 (12%)
I use a vasopressor only after assessment of preload dependency	371 (44%)
I use a vasopressor only after completed treatment of preload dependency	228 (27%)
I use a vasopressor regardless of preload dependency	121 (14%)
What is your main reason for increasing the dose of the vasopressor used?	
Diastolic arterial pressure target not reached	13 (2%)
Mean arterial pressure target not reached	568 (68%)
No arterial blood pressure response to the current dose	63 (8%)
Signs of organ dysfunction despite reaching the arterial blood pressure target	173 (21%)
Systolic arterial pressure target not reached	22 (3%)
What is your arterial blood pressure target for vasopressor therapy?	
A diastolic blood pressure > 40 mmHg	12 (1%)
A mean arterial pressure > 60–65 mmHg	584 (70%)
A mean arterial pressure > 70–75 mmHg	207 (25%)
A mean arterial pressure > 80–85 mmHg	24 (3%)
A systolic blood pressure > 100 mmHg	12 (1%)
Which patient’s factor(s) may encourage you to increase your arterial blood pressure target?	
Age	14 (2%)
History of chronic hypertension	662 (79%)
History of coronary artery disease	52 (6%)
None of them	102 (12%)
Value of central venous pressure	9 (1%)
When the patient does not respond to your current vasopressor therapy, what is your main reason for adding another vasopressor agent to the current therapy?	
A pre-defined maximum dose of the 1st choice vasopressor has been reached	119 (14%)
Although the pre-defined maximum dose of the 1st choice vasopressor has not been reached, previous increases in the dose of this vasopressor were ineffective	135 (16%)
By adding a second vasopressor although the pre-defined maximum dose of the 1st choice vasopressor has not been reached, I want to limit/reduce the side-effects of the first vasopressor	173 (21%)
I suppose that the mechanism of action of the first vasopressor is exhausted (e.g., adrenoceptors down regulation) and want to use a second one with an independent mechanism of action	213 (25%)
I want to use synergistic effects of two different mechanisms of action	199 (24%)
What is your main reason for reducing or stopping vasopressor therapy?	
Arterial blood pressure targets have been reached	463 (55%)
I am concerned by potential side effects of current vasopressor therapy	39 (5%)
Side effects of current vasopressor have occurred	15 (2%)
The patient’s clinical situation is improving even if the arterial blood pressure target has not been reached	296 (35%)
Vasopressor treatment is futile	26 (3%)
Which of the following statements fits best your opinion on norepinephrine use in the treatment of shock?	
Restoring mean arterial pressure with norepinephrine is usually associated with a decrease in systemic blood flow	69 (8%)
Restoring mean arterial pressure with norepinephrine is usually associated with a deterioration of renal function	9 (1%)
Restoring mean arterial pressure with norepinephrine is usually associated with a reduction in microcirculatory blood flow and/or tissue oxygenation	201 (24%)
Restoring mean arterial pressure with norepinephrine is usually associated with an increase in systemic blood flow	442 (53%)
Restoring mean arterial pressure with norepinephrine is usually associated with no change in systemic blood flow	118 (14%)

### Survey development

The questionnaire was developed by TWLS and JLT. The Research Committee of the ESICM endorsed the survey. It was not pretested beforehand. Data were collected automatically using SurveyMonkey Inc. (www.surveymonkey.com). No personal information was collected, and no log-in was required to participate. Completion or internal consistency of items was enforced by displaying an alert before the questionnaire was submitted and by highlighting mandatory but unanswered questions. It was not possible to review and change the given answers after submission. The 17 questionnaire items related to this study are provided in Tables 1 and 2.

The survey was announced on the ESICM website and was open for participation between November 2016 and April 2017. Members of the Cardiovascular Dynamics section of the ESICM were additionally encouraged to participate via an email linking to the survey sent to email addresses in ESICM’s membership database in November 2016 with two subsequent email reminders in February and March 2017. No incentives were offered for participation.

### Survey reporting

The methodology and results of the questionnaire are reported according to the Checklist for Reporting Results of Internet E-Surveys (CHERRIES) statement [18]. Ethical approval was not requested as this was a voluntary survey, and no individual patient data were collected.

### Experts’ recommendations

**Fig. 1:**
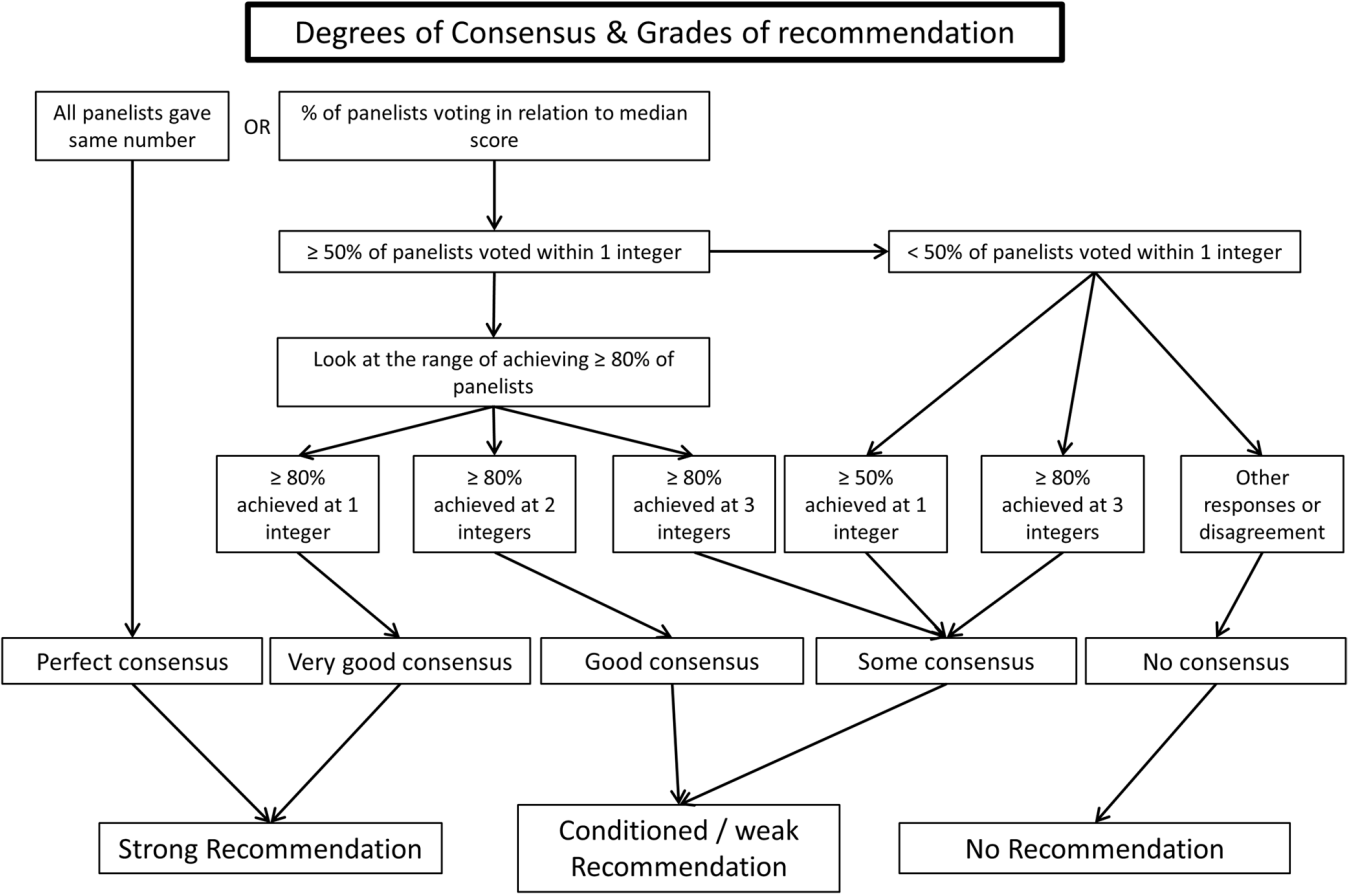
RAND algorithm. Method used to define the degree of consensus and grades of recommendations of the experts’ recommendations

Based on the analysis of the results, three authors (TWLS, IVDH and JLT) identified areas of interest and developed six questions, including sub-questions and approached a group of 34 experts who are active members of the Cardiovascular Dynamics Section of the ESICM, and who all have published research as first or last author in an international peer-reviewed journal in articles identified by the PubMed subject headings “vasopressor.” These experts were asked to formulate recommendations for the optimal use of vasopressors. Definitions of degree of consensus and grades of recommendations were based on the RAND algorithm (Fig. 1) [19]. Perfect consensus (all experts agreeing) and good consensus (≥ 80% agreement) were considered as strong grades of recommendation. Conditional recommendation was used when 70–80% of the experts agreed.

The questions posed to the experts are presented in Table 3. Sub-question 5e on the use of corticosteroids in refractory hypotension [20] was resent to the experts following the results of the ADRENAL [21] and APROCCHSS trials [22] to see whether these study results had changed their opinion.

**Table 3:** Questions to experts on vasopressor use

1. How should arterial blood pressure (ABP) be monitored in patients with septic shock?
2. What is the ideal time to start vasopressor therapy in treating septic shock?
a. Should hypovolemia be completely corrected first?
b. Which variable do you consider most helpful in deciding when to start vasopressor treatment?
3. Which vasopressor should be used as first choice?
a. Are there situations or patient categories in which a certain vasopressor should be preferred?
4. What is your target? Which variable and which value?
5. Concerning refractory hypotension [20]
a. What is your definition of refractory hypotension?
b. Do you accept a lower MAP when it is not possible to achieve the target MAP with high-dose vasopressors? In which situations?
c. When should a second vasopressor agent be considered? Which one?
d. Should it replace or be added to the first-choice vasopressor?
e. Should corticosteroids be used to reach the target?
6. What is your main reason for reducing or stopping vasopressor treatment?

#### Statistics

Data were evaluated as the total distribution of single answers and then divided according to the geographical area of respondents within Europe and outside Europe using descriptive statistics. Answers to the questionnaire items are reported as numbers (percentage). Contingency tables and corresponding Chi-square statistics are reported to describe the pairwise associations between selected demographic variables (European vs. non-European ESICM member, high-income vs. lower-income countries, intensive care unit (ICU) experience more vs. less than 5 years full time, intensive care (IC) as primary specialty vs. other specialties, and university hospital vs. non-university hospital) and the responses regarding vasopressor use. We used the World Bank definition of a “high-income country,” i.e., a per capita gross national income of $12,056 or more [23].

All descriptive and statistical analyses were performed in R (R studio version 1.1.453, running R version 3.5.0).

## Results

**Fig. 2:**
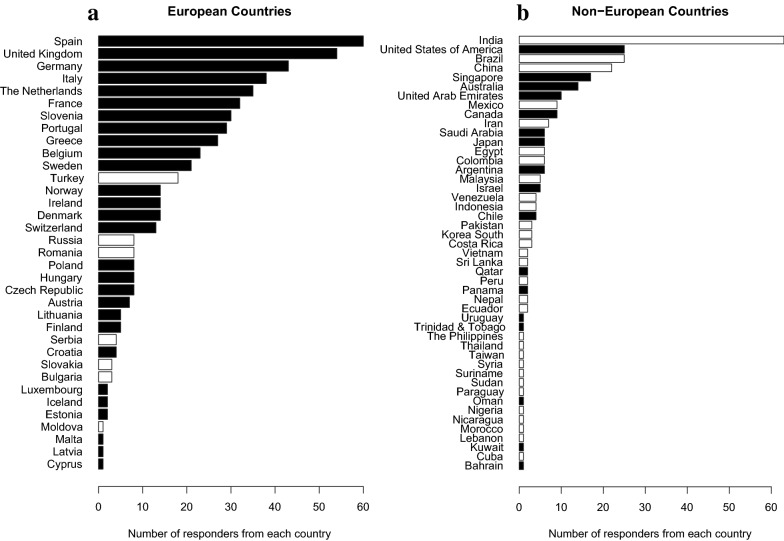
**a** Survey respondents from European countries. Number of survey respondents working in European countries. Black bars indicate high-income countries, and white bars lower-income countries. **b** Survey respondents from Non-European countries. Number of survey respondents working in Non-European countries. Black bars indicate high-income countries, and white bars lower-income countries

A total of 839 physicians from 82 countries participated in the survey. A response rate could not be calculated as the invitation to the survey was posted as a link on the ESICM open website. In addition, members of the CD section of the ESICM (*n* = 10,780 at the time of the survey) received an email invitation to participate. From these addressees, 3111 (29%) opened this email (according to Mail Chimp). Baseline characteristics of responders and their ICUs are presented in Table 1. Of the 839 participants, 546 (65%) were European (Fig. 2), 227 (27%) were from lower-income countries, and 353 (42%) were working in a university hospital. Four hundred and forty-five (53%) had more than 5 years of experience as an intensivist, and 545 (65%) had Intensive Care as their main specialty or activity area. All ten survey questions and answers of the physicians on arterial blood pressure and vasopressors are summarized in Table 2. Arterial blood pressure was always measured invasively by 707 (84%) of the participants. More non-European than European physicians (31% vs. 7.5%, *p* < 0.05), more respondents from lower-income countries than from high-income countries (37% vs. 8%, *p* < 0.001), and more IC specialists than non-intensivists (18% vs. 12%, *p* < 0.05) did not always measure ABP invasively. Norepinephrine was used by 816 (97%) respondents as the first-line vasopressor in septic shock, while more respondents from lower-income countries preferred a different vasopressor (6% vs. 1.5% from high-income countries, *p* < 0.001). Intensivists working in a university hospital were more likely to use another vasopressor than norepinephrine as their first-line treatment (4.5% vs. 1.4% of doctors working in non-university hospitals, *p* < 0.05). An insufficient MAP response to initial fluid treatment was the main trigger to initiate vasopressor administration as reported by 700 (83%). Early use of a vasopressor (despite/regardless of preload dependency) was preferred by 225 (26%) responders. A blood pressure target of MAP > 60–65 mmHg or DAP > 40 mmHg was chosen by 596 (71%) of respondents, with more respondents working in a university hospital preferring this target (75% vs. 68% of doctors working in non-university hospitals, *p* < 0.05). Six hundred and sixty-two (79%) participants modified their ABP target in patients with a history of chronic arterial hypertension. In addition, 19% of IC specialists considered reasons other than chronic hypertension (mostly non-patient related factors) as a trigger to increase their ABP target versus 26% of non-intensivists (*p* < 0.05). While the main reason for increasing the vasopressor dose was failure to reach the targeted blood pressure (68%), some respondents increased vasopressor doses for other reasons; e.g., signs of organ dysfunction despite reaching the MAP target. European-based intensivists and IC specialists more frequently chose to increase vasopressor dosages beyond reaching the target blood pressure (35% vs. 27% of non-Europeans, *p* < 0.05 and 37% vs. 30% of IC specialists, *p* < 0.05). There were no differences in any of the answers between experienced and less-experienced (< 5-year ICU experience) physicians.

The 34 experts agreed on 10 recommendations concerning arterial blood pressure and use of vasopressors and corticosteroids, 9 of which were strong (see Table 4). In addition, they recommended not to delay vasopressor treatment until fluid resuscitation has been completed, but rather start with norepinephrine early to achieve a target MAP of ≥ 65 mmHg, and to accept a lower MAP if it is sufficient to correct signs of hypoperfusion.

**Table 4:** Summary of the expert’s recommendations and its degree of consensus and grade of recommendation

Statement	Degree of consensus	Grade of recommendation
Blood pressure monitoring		
1. In patients with shock, arterial blood pressure should be monitored invasively and continuously via an arterial catheter	Perfect	Strong
Ideal moment to start vasopressor therapy in treating circulatory shock		
2. Vasopressors should be started early, before (complete) completion of fluid resuscitation	Reasonable	Conditional
3. MAP or the combination of MAP and DAP should be considered as trigger to start vasopressor treatment	Good	Strong
Vasopressor of first choice		
4. Norepinephrine should be used as vasopressor of first choice	Perfect	Strong
Target of vasopressor treatment		
5. The target of vasopressor therapy should be a MAP of 65 mmHg	Good	Strong
6. Lower MAPs are tolerated in case of refractory hypotension despite adequate fluid and vasopressor treatment	Good	Strong
Treatment options in refractory hypotension		
7. Adding a second vasopressor in case of refractory hypotension	Good	Strong
8. Using vasopressin or terlipressin as second vasopressor	Good	Strong
Reason to stop vasopressor treatment		
9. Vasopressor treatment should be reduced/stopped when the patient improves clinically, when side effects occur, or in case of ineffectiveness	Perfect	Strong
Use of steroids to reach target		
10. Steroids should be considered in septic shock	Good	Strong

Definitions of degree of consensus and grades of recommendations based on the RAND algorithm. All 34 experts in agreement defined a perfect consensus and experts ≥ 80% agreement defined good consensus; both were considered as strong recommendation. Reasonable consensus was defined as 70–80% agreement among experts, and the recommendation was considered to be conditional

## Discussion

Norepinephrine was reported to be the first-line vasopressor used to achieve MAP targets for almost all respondents to our online survey. Furthermore, a majority of respondents and experts would target an initial MAP of 65 mmHg or higher. These findings are in concordance with current guidelines for the management of sepsis and septic shock that recommend an initial target MAP of 65 mmHg and to titrate to individual requirements thereafter [8]. Notably, data from registries and major trials revealed that the average MAP in actual practice ranged between 75 and 80 mmHg. For example, in the SEPSISPAM trial, MAP was 75 mmHg in the low blood pressure group, whereas the prescribed target range was 65–70 mmHg [24]. Similarly, in the OVATION trial, half of the MAP measurements were above the targeted range [25]. This could suggest that healthcare professionals in the ICU used the higher blood pressures as a “safety-cushion” to prevent dipping below the target or that the vasopressor doses were not lowered when MAP improved. Recent retrospective analysis from 110 US hospitals shows that risks for mortality, AKI, and myocardial injury in septic patients progressively worsened at MAP thresholds lower than 85 mmHg [26].

Strikingly, the majority of respondents evaluate the effects of their initial resuscitation efforts based on their effects on blood pressure, whereas only 7% used cardiac output for this purpose. This is in line with previous studies [27, 28] but in contrast to the rational of fluid resuscitation which is to increase blood flow, i.e., cardiac output and oxygen delivery to ultimately improve tissue perfusion and oxygenation.

A large majority of physicians stated they would raise their ABP targets when the patient had a history of chronic arterial hypertension; this is also in line with current recommendations of the European consensus conference [2]. This strategy is based on alterations in autoregulation of organ perfusion occurring in hypertensive patients, although cerebral, hepatosplanchnic and renal autoregulation may be disturbed in the presence of severe systemic inflammation [29]. The SEPSISPAM trial found that targeting a higher MAP in septic patients with chronic arterial hypertension led to less requirement for renal replacement therapy [24]. On the other hand, a multicenter pilot randomized controlled trial reported that in patients aged ≥ 75 years, a lower MAP target (60–65 mmHg) was associated with a lower hospital mortality (13% vs. 60%, *p* = 0.03), while this was not true for younger patients [25]. Importantly, only 25 patients (8 deaths) were enrolled in the ≥ 75-year age-group so these results need to be interpreted with caution. A recent individual patient data meta‐analysis from two major trials comparing higher versus lower MAP targets revealed that higher MAP targets may be associated with a higher mortality, particularly when patients had been treated with vasopressors for > 6 h before inclusion [11]. Another cohort study on vasopressor use for severe arterial hypotension reported an average MAP of 75 mmHg and that ICU staff did not tailor vasopressor therapy to individual patient characteristics such as underlying chronic hypertension [30]. An option worth consideration is individualization of blood pressure targets, based on a “vasopressor challenge,” with return to the previous vasopressor dose if organ perfusion does not obviously improve while higher MAP levels were achieved, or if adverse effects such as atrial fibrillation or myocardial ischemia occur. The efficacy of this pragmatic strategy has not yet been confirmed by prospective studies, but has been tested in a recently completed study on early resuscitation in septic shock patients [31].

The choice of first-line vasopressor in our survey agrees with reports from Scandinavian and Canadian ICUs where norepinephrine was the first-line vasopressor used to achieve MAP targets [32, 33]. This is a significant change from an earlier survey where dopamine was the first-line vasopressor [34]. A large multicenter randomized controlled trial comparing norepinephrine versus dopamine [6], three meta-analyses [35–37], and subsequent guideline recommendations [7, 8] are likely to be the main contributors to this shift in practice. A recent retrospective analysis reported an increased mortality rate in septic shock patients managed with different vasopressors (predominantly phenylephrine) during a period of norepinephrine shortage in the USA [38, 39]. This implies that norepinephrine may be the vasopressor associated with the lowest mortality. Consequently, the 2016 SSC states that phenylephrine use should be limited until more research is available since its impact on clinical outcomes is uncertain [8].

The 2016 Surviving Sepsis Campaign suggests adding either vasopressin (up to 0.03 U min^−1^) (weak recommendation, moderate quality of evidence) or epinephrine (weak recommendation, low quality of evidence) to norepinephrine with the intent of raising MAP to target or adding vasopressin (up to 0.03 U min^−1^) (weak recommendation, moderate quality of evidence) to decrease norepinephrine dosage [8]. However, recent studies found no beneficial outcome effect from vasopressin [40] or terlipressin [41]. Angiotensin II has been studied as an additional vasopressor to maintain MAP in a recent randomized controlled trial in patients with vasodilatory shock [42]. Its exact place in the treatment of septic shock needs to be defined, but a subgroup analysis of the latter study suggests that patients with acute kidney injury requiring renal replacement may preferentially benefit from this treatment [43].

The timing to initiate vasopressor therapy varied in our survey; 44% of responders would start vasopressors after assessment of preload dependency, while 27% would use vasopressors only after complete correction of hypovolemia as assessed by preload dependency variables. The experts agreed with a conditional degree of consensus that vasopressors should be started before the completion of full fluid resuscitation. From the SSC guidelines, there is uncertainty about when vasopressors should be initiated in septic shock. After careful reading of the publication, it might be understood that vasopressors should be administered only after the initial fluid resuscitation (30 mL kg^−1^ of crystalloids within the first 3 h) [7]. This lack of clarity was criticized [44]. Data from the Australasian Resuscitation In Sepsis Evaluation (ARISE) trial showed that the median [IQR] volume of fluid administered before starting a vasopressor was 3.1 [2.3, 4.3] L [45].

Recently (after completion of our survey), the SSC proposed a new 1-hour bundle where vasopressors are recommended to be applied if the patient is hypotensive during or after fluid resuscitation to maintain MAP ≥ 65 mmHg [9]. Although it is not mentioned which indicator can be used to select patients who require vasopressors, this recommendation clearly indicates that early administration before complete fluid resuscitation is an option. Some studies reported that delay in initiation of vasopressor therapy was associated with an increased mortality risk in patients with septic shock [46, 47]. There are three potential reasons for this finding: early vasopressors could prevent the onset or progression of organ dysfunction by reaching the target MAP (as the main component of organ perfusion pressure) faster and by optimizing tissue perfusion [48, 49]. Earlier vasopressor therapy may represent a marker of the intensity of delivered care which could result in improved outcome. Finally, earlier vasopressor use could lead to a decrease in the amount of fluids administered [50], e.g., due to a redistribution of venous blood from unstressed to stressed volume (autotransfusion). However, retrospective data from almost 2900 patients from 24 hospitals in three countries suggest that starting vasoactive agents in the initial hour may be detrimental due to less fluids being given and that mortality was lowest when vasoactive agents were initiated 1–6 h after septic shock onset, with more than 1 L of fluids in the initial hour, more than 2.4 L from hours 1–6, and 1.6–3.5 L from 6 to 24 h [51]. In the ARISE trial, 50% of the patients received vasopressors within 4.4 h after hospital admission [45]. As these data reflect epidemiology rather than physiology, the optimal timing of vasopressor initiation needs to be studied in a personalized context.

In our survey, there was a discrepancy in the respondents’ opinion as to reasons why a second vasopressor should be added in patients with refractory hypotension, i.e., when a patient does not adequately respond to the initial vasopressor treatment. Only 14% of respondents cited a predefined maximum dose of the first vasopressor as the main reason. There is some support for this in the current literature as a post hoc analysis study found that vasopressor load and thresholds of dose have been related to mortality in septic shock [52]. This might be related to the occurrence of catecholamine-associated complications although the mortality associated with high-dose norepinephrine varies considerably. In a series of 324 patients with septic shock (average mortality rate 48%), patients who received norepinephrine doses ≥ 1 µg kg^−1^ min^−1^ had an extremely high (90%) mortality rate [53]. By contrast, in a series of 106 patients with severe septic shock who received ≥ 1 µg kg^−1^ min^−1^, the mortality rate was far lower (60%) [35]. Research is needed to identify clinically relevant thresholds for the consistency of guidelines and for design of future clinical trials [54].

Regarding the use of corticosteroids in refractory hypotension, 29/34 experts recommended its use despite the lack of strong evidence showing mortality benefit [55–57]. However, there is evidence that use of low-dose corticosteroids results in earlier shock reversal (i.e., reduced duration of vasopressor therapy with stable hemodynamics) in patients with septic shock unresponsive to fluid and vasopressor therapy [56–58]. Of note, no expert changed his/her mind after the results of the ADRENAL trial [21] became available, whereas two of the five experts with an initially negative attitude changed their opinion in favor of steroids after the results of the APROCCHSS trial [22].

In our survey, we received contradictory responses to the question regarding the change in cardiac output when restoring MAP with norepinephrine. Only 53% of physicians acknowledged that using norepinephrine to improve MAP might also result in an increase in systemic blood flow. Studies have shown increases in cardiac output through an increase in cardiac preload and cardiac contractility in patients with septic shock treated with norepinephrine [48, 59–62]. A recent systematic review has confirmed these findings [63]. Although 24% of responding physicians considered that restoring MAP with norepinephrine might result in a reduction in microcirculatory blood flow, this is not supported by recent studies showing improvements [49, 61, 64, 65], or no change [66–68] in microvascular perfusion in patients with septic shock when blood pressure was increased with norepinephrine. It appears that the effect of norepinephrine was dependent on the basal microvascular state, being beneficial only when the microcirculation was compromised.

Respondents had different opinions on how to measure blood pressure, MAP targets, dosing, timing, triggers for adding a second vasopressor, reasons for reducing the vasopressor dose, and stopping vasopressor treatment. This variation may be interpreted in two ways. Firstly, individual physicians may interpret the existing scientific evidence differently. For example, one physician may give more weight to a MAP target, while another may focus on signs of organ dysfunction. This is supported by the finding that 68% of respondents preferred MAP and 21% organ function markers as their target for vasopressor therapy. Secondly, the physicians may have interpreted the existing evidence in a similar manner, while the heterogeneity of septic shock drives the differences in treatment plans. These treatments may be adapted to individual patients based on their history, underlying disease, comorbidities, and response to treatment [69]. In clinical practice, a MAP target of 65 mmHg may be acceptable provided no other signs of hypoperfusion are present. If signs of hypoperfusion remain, the MAP target may need to be elevated. These nuances cannot be captured by a simple survey.

Although surveys are not at the top of the evidence-based pyramid, the results of this survey present useful information on contemporary practice and preferences regarding vasopressor therapy, obtained from responders from many European and non-European countries (Fig. 2). Non-European physicians more often used noninvasive techniques to measure ABP and less frequently considered other reasons than reaching the MAP target to increase the vasopressor dosage, such as persisting signs of organ dysfunction despite reaching MAP targets. These differences might reflect varying adoption rates of the Surviving Sepsis Campaign guidelines, or simply differences in available resources and local practices.

The experts’ opinions are based on the available evidence and their interpretation thereof for most of the questions, while its added value may especially lie in the questions where evidence is sparse. Furthermore, this work identified areas for future research as reflected by heterogeneous opinions.

The results of our survey can be used as a benchmark for interpreting studies stating usual or standard care in control groups of intervention trials. However, if the control group is treated (very) differently from what was reported in our survey, then external validity of results is diminished. Physicians are less swayed by the impact of an intervention when compared against a control intervention that is currently not considered as standard for treating patients. Furthermore, future trials can be designed to investigate changes against what is considered usual or standard care to increase the external validity. Another positive aspect of this survey is that it can be used to guide education, for example the need to avoid unnecessary fluid overload.

### Limitations

The methods used to invite individuals to respond to our survey did not allow us to calculate the exact response rate, which can be estimated to around 10% of all ESICM members. Nevertheless, our survey had by far the largest absolute number of respondents as compared to previous surveys on vasopressors (839 vs. 114, 171, and 202, respectively) [32–34]. Still, a response bias cannot be excluded. Results relate only to individuals who were willing to respond. External validity is therefore hampered. In addition, online surveys have limitations, including multiple responses by a single person. We did not use cookies or log-file/IP address analyses to prevent multiple responses. On the other hand, we assume that single persons are unlikely to spend time answering a simple survey more than once, and we are not aware if some institutions had higher representations among respondents than others. Furthermore, a survey may not reflect bedside practice rather than preferences, even in the institutions of the physicians answering the survey. In addition, questions and definitions used in our survey might have been interpreted differently by the respondents hampering their answers. Similarly, it should be noted that we currently have the third international consensus definition of sepsis [15], whereas most of the studies cited in the discussion were based on the criteria of the second definition.

## Conclusion

In conclusion, vasopressor use in critically ill patients with septic shock, as self-reported by individual physicians, is compliant with current guidelines. Experts recommended not to delay vasopressor treatment until fluid resuscitation is completed, but rather to start with norepinephrine early to achieve a target MAP of ≥ 65 mmHg. Future studies should focus on the implementation of current evidence on the early use of vasopressors, individualized hemodynamic targets, and patient outcomes [54]. A logical follow-up would be a systematic review on the use of vasopressors in critically ill adult patients with circulatory shock.

## Abbreviations

ESICM: European Society of Intensive Care Medicine
MAP: mean arterial pressure
ABP: arterial blood pressure
SSC: Surviving Sepsis Campaign
CHERRIES: Checklist for Reporting Results of Internet E-Surveys
ICU: intensive care unit
IC: intensive care
